# Pro-inflammatory cytokine-driven PI3K/Akt/Sp1 signalling and H_2_S production facilitates the pathogenesis of severe acute pancreatitis

**DOI:** 10.1042/BSR20160483

**Published:** 2017-04-28

**Authors:** Ying Liu, Ribin Liao, Zhanrong Qiang, Cheng Zhang

**Affiliations:** Department of Gastroenterology, The Second Affiliated Hospital of Guilin Medical University, Guilin 541000, People’s Republic of China

**Keywords:** cytokine, cell signaling pathways, hydrogen sulfide, intestinal motility, severe acute pancreatitis

## Abstract

Severe acute pancreatitis (SAP) is a disease usually associated with systemic organ dysfunction or pancreatic necrosis. Most patients with SAP suffer from defective intestinal motility in the early phase of the disease. Additionally, SAP-induced inflammation produces hydrogen sulphide (H_2_S) that impairs the gastrointestinal (GI) system. However, the exact mechanism of H_2_S in the regulation of SAP is yet to be elucidated. In the present paper, we used a rat model of SAP to evaluate the role of H_2_S on intestinal motility by counting the number of bowel movements and investigating the effect of H_2_S on inflammation. We treated colonic muscle cells (CMCs) with SAP plasma, tumour necrosis factor-α (TNF-α) or interleukin-6 (IL-6) and measured the expressions of H_2_S-producing enzymes cystathionine-γ-lyase (CSE), cystathionine-β-synthase (CBS) and *Sp1* and PI3K/Akt by using quantitative PCR, Western blotting and immunohistochemical detection. We used the PI3K inhibitor LY294002 and the siRNA si-*Sp1* to suppress the activity of the PI3K/Akt/*Sp1* signalling pathway. We found that, in the SAP rat model, H_2_S facilitated an inhibitory effect on intestinal motility and enhanced the inflammatory response caused by SAP (*P*<0.05). The expressions of CSE and CBS in CMCs were significantly increased after treatment with TNF-α or IL-6 (*P*<0.05). Blocking the PI3K/Akt/*Sp1* pathway remarkably inhibited the synthesis of CSE and CBS. Our data demonstrated that H_2_S plays a vital role in the pathogenesis of SAP and that SAP is modulated by inflammation driven by the PI3K/Akt/*Sp1* signalling pathway.

## Introduction

The severe form of acute pancreatitis (AP) is classified as severe AP (SAP) and is associated with a mortality rate as high as 30% [1]. Management of SAP is complicated due to the lack of knowledge of the pathogenesis, which increases the uncertainties in the prognosis and thus impedes the effective therapy [2]. SAP presents a serious gastrointestinal (GI) tract or ileal obstruction during the early stage of the disease [3]. However, conventional treatment often overlooks the pivotal role of the intestine in SAP. Recent evidence suggests that bacterial translocation and intestinal sepsis play key roles in SAP [4,5]. Thus, novel therapies for SAP aiming at limiting intestinal injury are being developed, including use of several antioxidant and anti-cytokine agents [6–8]. Nevertheless, the definite relationship between SAP and the intestine remains obscure.

Hydrogen sulphide (H_2_S), at normal temperature, is a colourless gas with a strong odour and performs many physiological functions [9]. It is predominantly produced by the activation of cystathionine-γ-lyase (CSE), cystathionine-β-synthase (CBS) and 3-mercaptopyruvate sulphurtransferase (3-MST). H_2_S is present in many tissues of the body and initiates pharmacophysiological responses in most organ systems [10,11]. In the recent decade, particular interest in the role of H_2_S in the GI tract is emerging based on the finding that it is produced both by GI tissues and generated in large quantities by bacterial flora in the lumen of the gut [12]. The production of H_2_S has been shown to inhibit GI motility in a fish model [12]. Interestingly, the synthesis of H_2_S induced in the GI system also serves as an inhibitor of the inflammatory response [13].

Although three enzymes are involved in the synthesis of H_2_S, CSE is reported to be the major enzyme in the production of H_2_S in peripheral tissues [14]. Previous studies have implicated a role for PI3K/Akt/*Sp1* signalling in the regulation of CSE [15]. In the present study, we investigated the role of H_2_S and its possible role in SAP in a rat model.

## Materials and methods

### Chemicals, cell cultures and animals

Antibodies against CSE, CBS, PI3K, pPI3K, Akt, p-Akt, *Sp1* and GAPDH were purchased from Abcam. PI3K inhibitor LY294002 was purchased from Sigma. Rat colonic muscle cells (CMCs) were separated from the mucous membrane of the proximal colon and cultured in a solution containing 0.15% collagenase II, 0.1% trypsin inhibitor and 0.25% FBS at 37°C for subsequent experiments. Identification of CMCs was performed with immunofluorescent validation of α-actin, as described below. Adult male Wistar rats (weighing 200–250 g) were provided by the Guilin Medical University Experiment Center and maintained in cages at room temperature (20–25°C) with constant humidity (55 ± 5%) with free access to food and water *ad libitum*. All animal experiments were conducted in accordance with the Institutional Animal Ethics Committee and Animal Care Guidelines for the Care and Use of Laboratory Animals of Guilin Medical University.

### Induction of SAP in a rat model

Thirty rats were selected and randomly divided into three groups (ten in each group): the sham group where rats underwent a laparotomy and flip of duodenum and pancreas like the animals in the SAP group but without being injected the corresponding agents, the SAP group where rats underwent a laparotomy and were then injected with 5% sodium taurocholate into the bile and the ducts using a microinjection pump (1 ml/kg, 0.1 ml/min) for 10 min followed by suturing of the incision and the SAP + PAG (propargylglycine) group where rats underwent the same surgical procedures as the SAP group but with 30 μmol/kg PAG (inhibitor of CSE) injected into the bile and the pancreatic ducts in addition to the sodium taurocholate. All the rats were subcutaneously injected with 10 ml of normal saline and were then fasted for 24 h before subsequent experiments.

### Treatment of CMCs with TNF-α and IL-6

To explore the role of inflammation in modulating CSE and associated pathways, CMCs were administrated with 5% plasma from rats with SAP, 10 μg/l of tumour necrosis factor-α (TNF-α) or 10 μg/l of interleukin-6 (IL-6). The expressions of CSE, CBS, *Sp1* and PI3K/Akt were then detected using real-time quantitative PCR (RT-qPCR) and Western blotting.

### Knockdown of *Sp1* gene in CMCs

A specific *Sp1* siRNA (5′-GCAACAUGGGAAUUAUGAATT-3′) was constructed by GenePharma (Shanghai) and ligated into the shuttle vector pGL3. Transfection was conducted using Lipofectamine 2000 (Invitrogen), according to the manufacturer’s instructions. CMCs were divided into four groups: the NC group with healthy CMCs, the LY294002 group with healthy CMCs that were incubated with 5.0 μmol/l of LY294002 for 24 h, the vector group with healthy CMCs that were transfected with the blank pGL3 vector and the si-*Sp1* group with healthy CMCs that were transfected with the pGL3 vector and *Sp1* siRNA. Each treatment was represented by three replicates.

### Measurement of intestinal motility

The intestinal motilities of experimental rats were represented by the number of bowel movements. The measurements were performed 1 h before SAP induction and 4, 8, 12, 16, 20, 24 h after SAP induction, by two independent observers who were blind to the experimental design.

### Detection of the SAP-induced inflammatory response

After the intestinal motility measurements were completed, rats were sacrificed and plasma was collected. The inflammatory cytokines that were produced, including TNF-α and IL-6 were measured using CUSABIO ELISA kits, according to the manufacturer’s instructions.

### Immunohistochemical detection

For the immunohistochemical assay, paraffin sections of colonic muscle tissues from rats in the different groups were placed in 60°C for 2 h, followed by incubation with dimethylbenzene for dewaxing. The sections were then hydrated with different concentrations of alcohol (95% for 2 min, 85% for 2 min and 75% for 5 min) and washed with ddH_2_O for 2 min. Sections were then fixed using 3% H_2_O_2_ for 15 min and washed with PBS three times. They were then incubated with primary antibodies against CSE (Abcam, diluted 1:200) and CBS (Abcam, diluted 1:500) at 37°C for 30 min and incubated at 4°C overnight. After three cycles of 0.01 M PBS washes, 5 min for each cycle, the secondary antibody (1:200) was added to the sections and placed at 37°C for 30 min followed by another five cycles of PBS washes. HRP-labelled avidin was then added and incubated with the sections at 37°C for 30 min before addition of DAB. The reaction was kept for 3–10 min and then stopped by ddH_2_O. Slides were re-stained using haematoxylin and dehydrated. Finally, the slides were scanned using an Aperio ScanScope GL (Aperio Technologies, Vista, CA, U.S.A.) at 200× magnification.

### RT-qPCR

Whole RNA was extracted from tissues or cells in the different groups using RNA simple Total RNA Kit, according to the manufacturers’ instructions (Cat. No. DP419, TIANGEN, Beijing, China). *GAPDH* was selected as the internal reference gene. cDNA templates were prepared from total RNA using Super M-MLV Reverse Transcriptase (Cat. No. RP6502, BioTeke, Beijing, China), and the RT-qPCR reaction mixture with a final volume of 20 μl contained 10 μl of SYBR Premix Ex Taq II, 0.5 μl of each primer set (Supplementary Table S1), 1 μl of the cDNA template and 8 μl of RNase-free H_2_O. Thermal cycling parameters for amplification were as follows: a denaturation step at 95°C for 10 min, followed by 40 cycles at 95°C for 10 s, 60°C for 20 s and 72°C for 30 s. Relative expression levels of targeted genes were calculated with Data Assist Software version 3.0 (Applied Biosystems/Life Technologies), according to the expression 2^−ΔΔ*C*^^*t*^.

### Western blotting assay

Whole protein was extracted from the different cells using the Total Protein Extraction Kit, according to the manufacturer’s instructions (Cat. No. WLA019, Wanleibio, China). GAPDH was used as an internal reference protein. Concentrations of protein samples were determined using the BCA method and 40 μg of protein was subjected to 10% SDS/PAGE. After transferring targeted proteins on to PVDF sheets, the membranes were washed with TTBS for 5 min and then incubated with skimmed milk powder solution for 1 h. Primary antibodies against CSE (Abcam, diluted 1:500), CBS (Abcam, diluted 1:1000), PI3K (Abcam, diluted 1:2000), pPI3K (Abcam, diluted 1:1000), Akt (Abcam, diluted 1:2000), p-Akt (Abcam, diluted 1:1000), *Sp1* (Abcam, diluted 1:2500) or GAPDH (1:2000) were incubated with membranes at 4°C overnight. After additional four washes using TTBS, the secondary HRP IgG antibody (1:5000) was added and incubated with the membranes for 45 min at 37°C. After six washes with TTBS, the blots were developed using Beyo ECL Plus reagent and the results were recorded in the Gel Imaging System. The relative expression levels of GREM1 and GLI3 in different samples were calculated with Gel-Pro-Analyzer (Media Cybernetics, U.S.A.).

### Immunofluorescent assay

The distribution and expression of CSE after *Sp1* knockdown was detected with immunofluorescent microscopy. In brief, the treated cells were seeded into 14-well chambers, washed with PBS, fixed with 4% paraformaldehyde for 15 min. Then the cells were permeabilized with 0.5% Triton X-100 for 30 min. The cells were washed with PBS for three cycles, 5 min for each cycle and were blocked in 10% goat serum for 15 min. Primary rabbit polyclonal antibody to CSE (Abcam, diluted 1:50) was then added and the cells were incubated overnight at 4°C in 1% goat serum. Staining was performed by incubating the cells with Alexa Fluor 594–conjugated secondary antibodies for 1 h at room temperature. After incubation with the secondary antibody, cells were washed and then stained with DAPI for 5 min at room temperature. After three cycles of 5-min washes with PBS buffer, the slides were fixed and imaged with the fluorescent microscope at 200× magnification.

### Statistical analysis

All the data were expressed in the form of mean ± S.D. Comparison of means among multiple groups was done by one-way ANOVA. Tukey test was provided where equal variances were assumed, while Tamhane’s T2 test was used where equal variances were not assumed. Statistical significance was defined as *P*<0.05. All graphs were plotted and statistical analyses performed using GraphPad Prism 6 (GraphPad Software, San Diego, CA).

## Results

### H_2_S is involved in the suppression of intestinal motility

**Figure 1:**
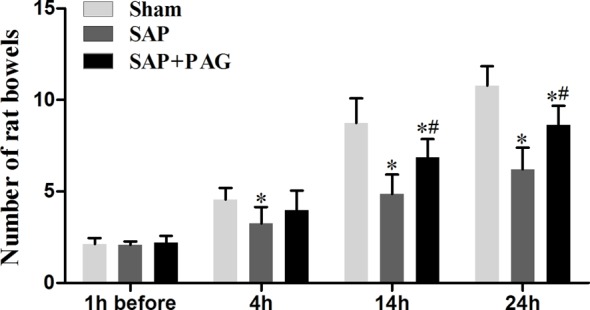
The number of bowels over different time periods. The number of bowel movements. Intestinal motility was determined by counting the number of bowel movements. *, significantly different from the sham group, *P*<0.05. #, significantly different from the SAP group, *P*<0.05 (*n*=10, each group).

The number of bowel movements in rats from the different groups was recorded to evaluate intestinal motility. As shown in Figure 1, before SAP induction, there was no difference in the number of bowel movements among the different groups. However, after SAP was induced, the number of bowel movements in the SAP group was significantly reduced compared with that in the sham (*P*<0.05) group. To elucidate whether the loss of intestinal motility was due to the production of H_2_S, rats were administered with PAG, which inhibits the production of H_2_S. We found that the suppression of H_2_S by PAG significantly increased intestinal motility, compared with the SAP group (*P*<0.05), suggesting a critical role for H_2_S in intestinal motility in rats with SAP.

### H_2_S is associated with SAP-induced up-regulation of the inflammatory response and the synthesis of CSE and CBS

**Figure 2:**
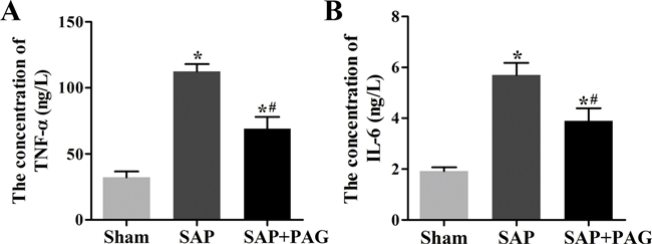
Expressions of inflammatory cytokines. Expressions of inflammatory cytokines by ELISA. (**A**) TNF-α. (**B**) IL-6. Concentrations of TNF-α and IL-6 in plasma of rats were increased after SAP induction. Suppression of H_2_S impeded the levels of inflammatory cytokines. *, significantly different from the sham group, *P*<0.05. #, significantly different from the SAP group, *P*<0.05 (*n*=10, each group).

**Figure 3:**
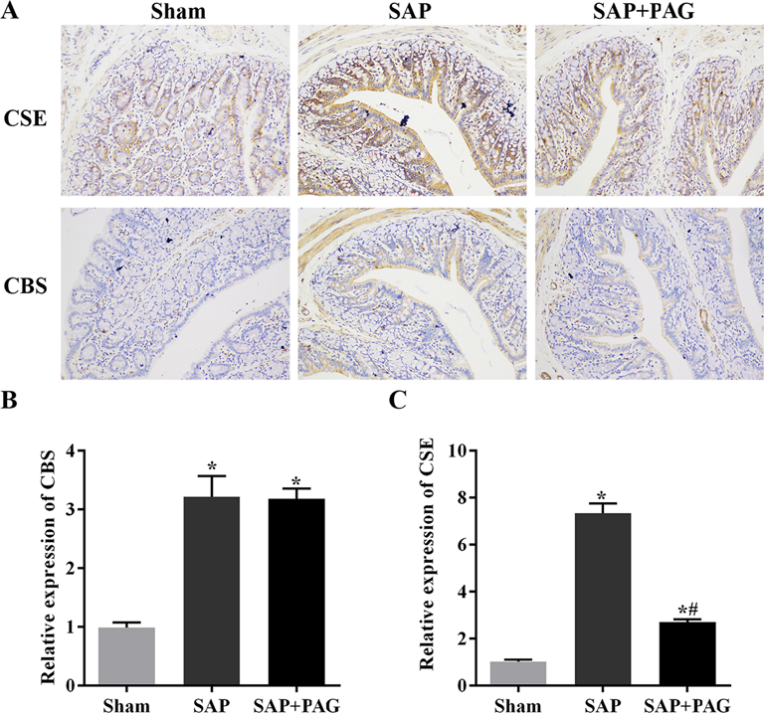
H_2_S synthesizing enzymes are induced by SAP. (**A**) Immunohistochemical detection. (**B**) Levels of CBS by quantitative RT-PCR. (**C**) Levels of CSE by quantitative RT-PCR. Images of immunohistochemical detection and quantitative RT-PCR were used to validate the expression of CSE and CBS. SAP significantly increased the expressions of CSE and CBS. PAG down-regulated the levels of CSE. *, significantly different from the sham group, *P*<0.05. #, significantly different from the SAP group, *P*<0.05 (*n*=10, each group).

Since H_2_S enhances inflammation, we tested the levels of inflammatory cytokines in the plasma of the rats using ELISA. Of note, expressions of TNF-α and IL-6 were dramatically increased in association with the SAP induction (Figure 2). Moreover, the decrease in H_2_S impaired the secretion of TNF-α and IL-6. In a similar fashion, SAP promoted the synthesis of CSE and CBS, whereas the suppression of H_2_S by PAG reduced the levels of CSE and CBS (Figure 3A-C), indicating that H_2_S plays a favourable role in the inflammatory response induced by SAP.

### PI3K/Akt-mediated *Sp1* signalling pathway is activated by SAP

**Figure 4:**
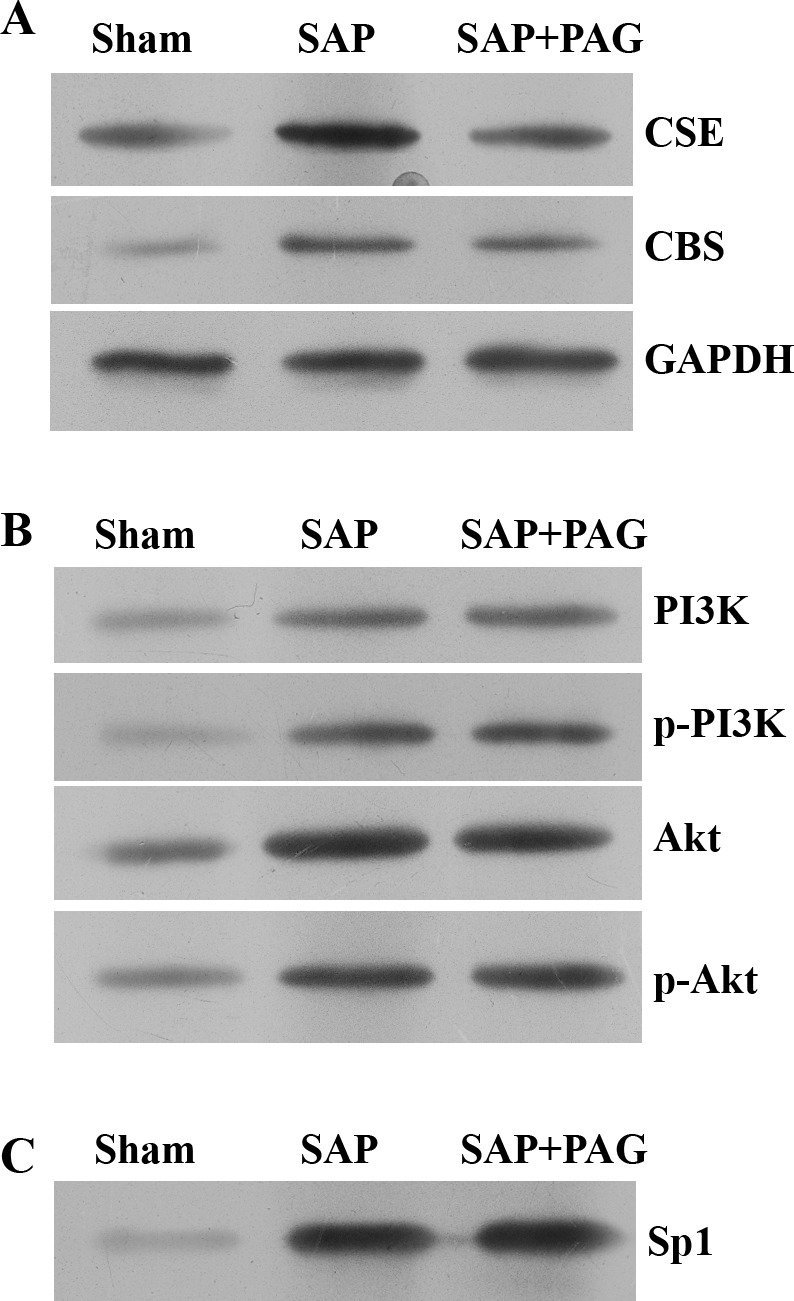
SAP activated *Sp1* signalling pathway. The expressions of CSE, CBS and PI3K/Akt/*Sp1* in plasma of rats by Western blotting. (**A**) The expressions of CSE, CBS proteins. (**B**) The expressions of PI3K, p-PI3K, Akt, p-Akt. (**C**) The levels of *Sp1* protein. GAPDH was set as inner reference and five samples in each group were detected.

To study the molecular mechanism of H_2_S in SAP, we investigated the signalling pathway of PI3K/Akt-mediated *Sp1*. Similar to the results from Figure 3B, Western blotting data showed that the expressions of CSE and CBS proteins in the SAP group were elevated compared with those in the sham group, but were reduced after H_2_S was inhibited in the SAP + PAG group (Figure 4A). Notably, phosphorylation levels of PI3K and Akt, were dramatically increased in the SAP and SAP + PAG groups, (which then up-regulated the expression of *Sp1*), compared with the sham group. There was no significant difference in PI3K/Akt/*Sp1* expressions between the SAP and SAP + PAG groups, suggesting a potential upstream regulator of PI3K/Akt/*Sp1* that produces H_2_S, through the modulation of CSE (Figure 4B,C).

### SAP induced the production of H_2_S by initiating the inflammatory response

**Figure 5:**
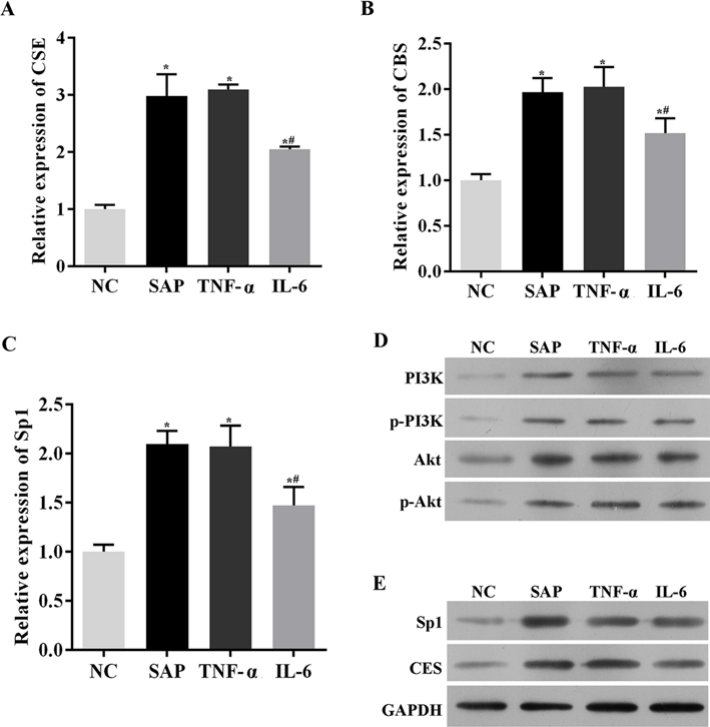
Plasma from SAP rats, TNF-α or IL-6 promoted production of H_2_S. The expressions of inflammatory cytokines in CMCs treated with 5% plasma from rats with SAP, TNF-α or IL-6. (**A**) Relative fold of CSE by qRT-PCR. (**B**) Relative fold of CBS by qRT-PCR. (**C**) Relative fold of *Sp1* by qRT-PCR. (**D**) The expressions of PI3K, pPI3K, Akt, p-Akt by Western blotting. (**E**) The expressions of *Sp1* by Western blotting. GAPDH was set as an inner reference. *, significantly different from the sham group, *P*<0.05. #, significantly different from the SAP group, *P*<0.05 (*n*=3, each group).

**Figure 6:**
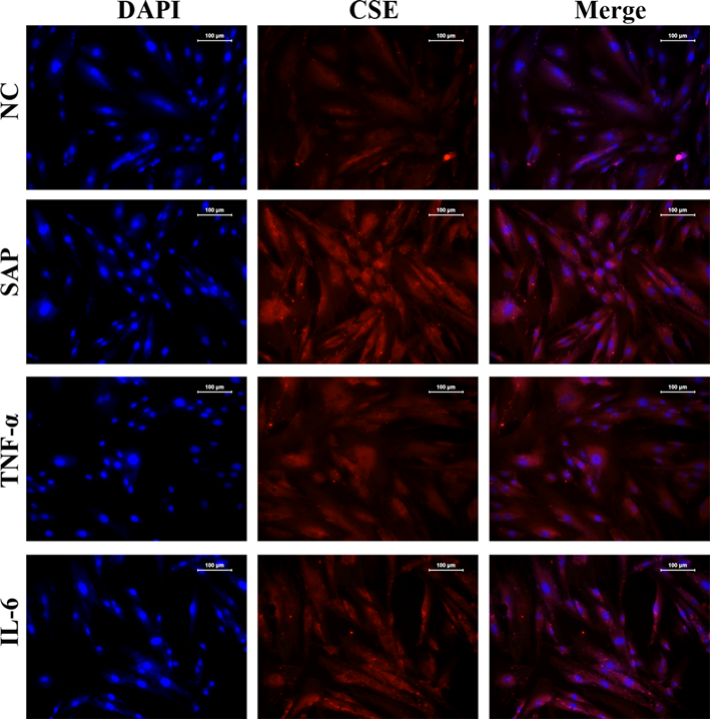
Plasma from SAP rats, TNF-α or IL-6 induced expression of CSE. Expression and distribution of CSE in CMCs treated with SAP plasma, TNF-α or IL-6 by immunohistochemical assay (200× magnification).

We used CMCs to explore the interaction between the inflammatory response and H_2_S production. We found that treating the CMCs with 5% plasma from SAP rats, TNF-α or IL-6, all contributed to the significantly increased expressions of CSE and CBS, compared with the NC group (*P*<0.05) (Figure 5A,B). Also, PI3K/Akt/*Sp1* signalling in CMCs was significantly activated after the treatment with 5% plasma from SAP rats, TNF-α or IL-6 compared with the NC group (*P*<0.05) (Figure 5C–E). The results confirmed our hypothesis that H_2_S production was induced by inflammatory cytokines associated with SAP symptoms. Considering that CSE is the major H_2_S-producing enzyme in peripheral tissues, the expression and distribution of the enzyme was further detected with an immunohistochemical assay (Figure 6), and the results were consistent with our RT-qPCR data in Figure 5A. Together, these results suggest that inflammatory cytokines, IL-6 or TNF-α increase the expression of H_2_S and CSE, in CMCs.

### SAP up-regulated the production of H_2_S via PI3K/Akt/*Sp1*l signalling pathway

**Figure 7:**
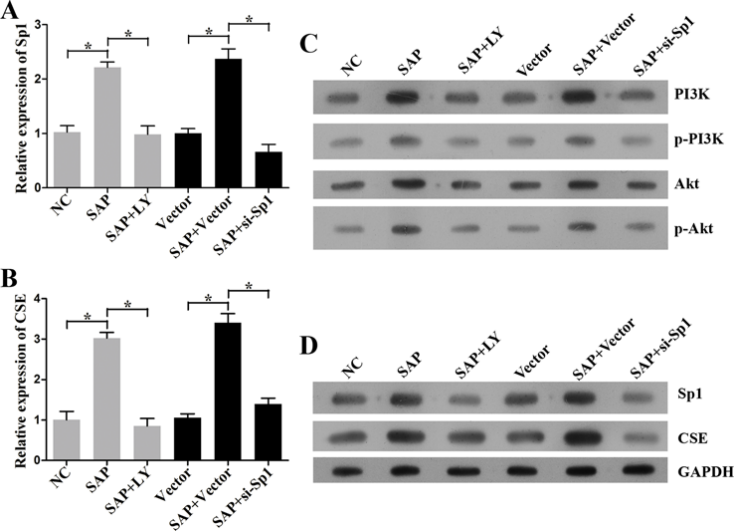
Expressions of CSE and levels of PI3K/Akt/*Sp1* in CMCs treated with LY294002 and si-*Sp1*. (**A**) The levels of *Sp1* by qRT-PCR. (**B**) The level of CSE by qRT-PCR. (**C**) The expressions of PI3K, pPI3K, Akt, p-Akt by Western blotting. (**D**) The expressions of *Sp1* by Western blotting. Inhibition of PI3K/Akt/*Sp1* signalling suppressed the expression of CSE. *, *P*<0.05, significantly different between the two groups (*n*=3, each group).

**Figure 8:**
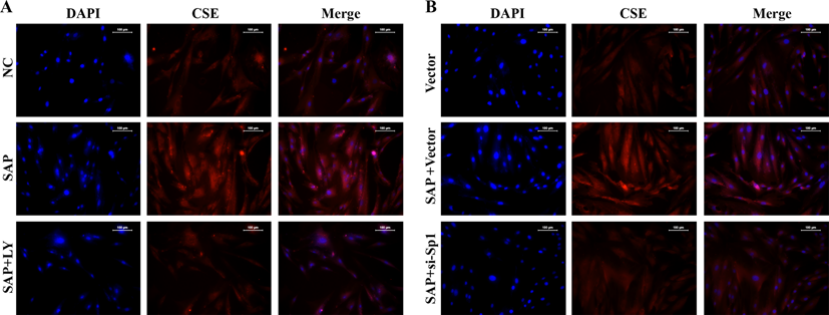
Expressions of CSE in CMCs treated with LY294002 and si-*Sp1* detected by immunohistochemical assay. Expression and distribution of CSE in SAP model was limited by administration of (**A**) PI3K inhibitor LY294002 and (**B**) *Sp1* siRNA as detected by immunohistochemical assay (200× magnification).

To validate that the PI3K/Akt-mediated *Sp1* modulates CSE in CMCs, we blocked the expression of PI3K with either an inhibitor, LY294002 or specific siRNA, si-*Sp1* vector. Our data showed that the activation of PI3K/Akt/*Sp1* signalling was impeded in CMCs with the treatment of LY294002 or si-*Sp1* (Figure 7A,C,D). Moreover, the suppression of PI3K/Akt/*Sp1* signalling pathway by LY294002 or si-*Sp1* also down-regulated the levels of CSE in CMCs treated with plasma from SAP rats (Figure 7B,D). In addition, the immunohistochemical analysis revealed that blocking PI3K/Akt/*Sp1* inhibited CSE synthesis after the induction of SAP (Figure 8A,B). All together, our data demonstrated that the expression of CSE is associated with PI3K/Akt/*Sp1* and suggested that the production of H_2_S is induced by SAP via the activation of the PI3K/Akt/*Sp1* signalling pathway.

## Discussion

The trans-sulphuration pathway is an important mechanism, by which cells provide cysteine to synthesize the cellular redox-controlling molecules and protect against damages induced by reactive oxygen species (ROS) [16]. Deficiencies in the trans-sulphuration pathway result in the overproduction of pro-inflammatory factors and cause a chronic inflammatory process, associated with the development of tumours and various diseases [16]. H_2_S is the third member of the gasotransmitter family and is endogeneously synthesized by CSE and CBS in the trans-sulphuration pathway [17]. H_2_S is increasingly recognized as a functionally relevant mediator with a number of physiological functions and abnormal production of H_2_S has been demonstrated to be associated with inflammation in mammalian tissues [18,19]. However, the interaction between H_2_S production and inflammation is complicated. In a study by Wallace et al. [13], the authors concluded that H_2_S played a protective role in the GI system by inhibiting the production of TNF-α and leucocyte adherence to the vascular endothelium. In contrast, Tamizhselvi et al. [19] reported that H_2_S is an inducer of the inflammatory response in rats with AP. Thus, the exact function and mechanism of H_2_S in the onset of SAP remains unknown. In the current study, the role of H_2_S in the development of SAP was further studied by targeting its effect on inflammation and intestinal motility.

Consistent with numerous previous studies, the induction of SAP initiates the expression of CSE and CBS [19–21], that leads to the production of H_2_S. Nevertheless, it was surprising to find that the augmented release of H_2_S in the current study was not only associated with the suppression of intestinal motility but also with the suppression of inflammation. After inhibiting the activity of CSE *in vivo* by PAG, which suppressed the production of H_2_S, the levels of TNF-α and IL-6 in the plasma were significantly increased. These results suggested an association between H_2_S and inflammatory cytokines. Therefore, CMCs isolated from rats were incubated with SAP plasma, TNF-α and IL-6 to investigate the role of H_2_S in SAP. We found that SAP plasma, TNF-α and IL-6, all induced the expression of CSE and CBS and enhanced the production of H_2_S. This finding is in agreement with previous reports that H_2_S plays a pro-inflammatory role in SAP [19–21].

In addition to the the reduced production of H_2_S, the intestinal motility of rats with SAP were remarkably inhibited, consistent with previous reports regarding the effect of H_2_S on the GI system [12,22,23]. The motility of the intestinal tract depends on the contraction of smooth muscles, which are regulated by ATP-sensitive K^+^ (K_ATP_) channels [24,25]. Typically, in smooth muscle cells, opening of the K_ATP_ channels will hyperpolarize the cell membrane, inactivate voltage-dependent L-type Ca^2+^ channels, and result in cell relaxation and blood vessel dilation by reducing intracellular free Ca^2+^ concentration [26]. As was previously reported, activation of K_ATP_ channels can be initiated by endogeneous and exogenous production of H_2_S [27–29]. Although cell membrane potential was not tested in the current study due to the limitation of experimental instruments, it is reasonable to speculate that the H_2_S produced in SAP patients was first induced by an inflammatory response and subsequently suppressed the colonic motility by activating the opening of K_ATP_ channels. Therefore, application of H_2_S in clinics should depend on a comprehensive assessment of the toxicity and side effects of the agent.

Underlying the inflammatory pathway associated with H_2_S production, the expression of *Sp1*, a critical regulator of CSE, was inhibited by a specific siRNA in our study. It is commonly recognized that the production of H_2_S is catalysed by CSE, CBS and 3-MST, of which CSE is reported to be the major H_2_S-producing enzyme in peripheral tissues [14]. A previous study showed that CSE expression is regulated by PI3K/Akt pathways via *Sp1* [15]. Given the multiple functions of the PI3K/Akt pathway in diverse biological processes including inflammation, the possibility of PI3K/Akt pathway in modulating H_2_S production in SAP was assessed. Previous work showed that both IL-6 and TNF-α contributed to the activity of PI3K/Akt pathway through multiple mechanisms [30, 31]. Based on our data, both SAP-induced inflammation *in vivo* and administering cytokines *in vitro* initiated the activation of the PI3K/Akt pathway. Moreover, the up-regulated activities of PI3K and Akt were shown to increase the synthesis of CSE through the regulation of the *Sp1* gene [15]. Partially consistent with the previous studies [32], CMCs treated with the PI3K inhibitor LY294002 inhibited *Sp1* activity and suppressed CSE synthesis. However, contrary to existing hypotheses, knockdown of *Sp1* also inhibited the phosphorylation of PI3K and Akt, the upstream regulators of *Sp1* [15]. Furthermore, knockdown of *Sp1* gene dramatically decreased the expression of CSE, which led to the suppression of H_2_S production, in spite of its effect on PI3K/Akt signalling. We concluded that the change in activity in PI3K/Akt/*Sp1* signalling is closely associated with the effect of H_2_S in SAP and this underlying mechanism needs to be further explored.

In conclusion, the current study demonstrated that H_2_S played an inhibitory role in intestinal motility of rats with SAP and promoted an inflammatory response during SAP. The production of H_2_S was induced by the inflammation-mediated activation of the PI3K/Akt/*Sp1* pathway. Our preliminary data indicate a role of H_2_S in the pathogenesis of SAP and provide potential leads for the discovery of a novel treatment against SAP.

## Abbreviations

Akt: Protein kinase B
AP: acute pancreatitis
CBS: cystathionine-β-synthase
CMC: colonic muscle cell
CSE: cystathionine-γ-lyase
GI: gastrointestinal
IL-6: interleukin-6
K_ATP_: ATP-sensitive K^+^
PAG: propargylglycine
PI3K: Phosphoinositide 3-kinase
RT-qPCR: real-time quantitative PCR
SAP: severe acute pancreatitis
*Sp1*: Specificity protein 1
TNF-α: tumour necrosis factor-α
3-MST: 3-mercaptopyruvate sulphurtransferase
